# Intensity of metastasis screening and survival outcomes in patients with breast cancer

**DOI:** 10.1038/s41598-021-82485-w

**Published:** 2021-02-02

**Authors:** Jong-Ho Cheun, Jigwang Jung, Eun-Shin Lee, Jiyoung Rhu, Han-Byoel Lee, Kyung-Hun Lee, Tae-Yong Kim, Wonshink Han, Seock-Ah Im, Dong-Young Noh, Hyeong-Gon Moon

**Affiliations:** 1 Department of Surgery, Seoul National University College of Medicine, 101 Daehak-ro, Jongno-gu, Seoul, 03080 Korea; 2 Department of Surgery, College of Medicine, The Catholic University of Korea, Seoul, Korea; 3 Department of Internal Medicine, Seoul National University College of Medicine, Seoul, Korea; 4 Cancer Research Institute, Seoul National University, Seoul, Korea; 5 Genomic Medicine Institute, Medical Research Center, Seoul National University College of Medicine, Seoul, Korea

**Keywords:** Breast cancer, Metastasis

## Abstract

Previous randomized trials, performed decades ago, showed no survival benefit of intensive screening for distant metastasis in breast cancer. However, recent improvements in targeted therapies and diagnostic accuracy of imaging have again raised the question of the clinical benefit of screening for distant metastasis. Therefore, we investigated the association between the use of modern imaging and survival of patients with breast cancer who eventually developed distant metastasis. We retrospectively reviewed data of 398 patients who developed distant metastasis after their initial curative treatment between January 2000 and December 2015. Patients in the less-intensive surveillance group (LSG) had significantly longer relapse-free survival than did patients in the intensive surveillance group (ISG) (8.7 vs. 22.8 months; p = 0.002). While the ISG showed worse overall survival than the LSG did (50.2 vs. 59.9 months; p = 0.015), the difference was insignificant after adjusting for other prognostic factors. Among the 225 asymptomatic patients whose metastases were detected on imaging, the intensity of screening did not affect overall survival. A small subgroup of patients showed poor survival outcomes when they underwent intensive screening. Patients with HR-/HER2 + tumors and patients who developed lung metastasis in the LSG had better overall survival than those in the ISG did. Highly intensive screening for distant metastasis in disease-free patients with breast cancer was not associated with significant survival benefits, despite the recent improvements in therapeutic options and diagnostic techniques.

## Introduction

Breast cancer is the most frequently diagnosed cancer and the leading cause of cancer-related death among women^1^. Despite improved overall survival among patients with breast cancer^2^, a significant number of patients eventually develop distant metastasis after initial treatment^3^. The diagnosis of distant metastasis in patients with breast cancer is clinically and psychologically important because the presence of metastasis results in a shift of disease-free status into incurable stage IV status.

Current major guidelines recommend against the use of routine imaging to detect distant metastasis in asymptomatic patients with breast cancer^4,5^. These recommendations are based on the findings of randomized trials that showed no survival or quality-of-life benefits on routine intensive imaging studies for breast cancer^6–8^. A recently updated systematic review of the randomized trials showed that regular physical examination and yearly mammograms are as effective as highly intensive imaging considering overall survival^9^. Thus, intensive screening for distant metastasis does not provide survival benefit but rather increases the risk of extending the duration of toxic treatment, as intensive screening might result in the earlier detection of metastatic lesions^6,7^.

However, the above-mentioned randomized trials were conducted nearly three decades ago when treatment strategies for resectable breast cancer were substantially different from those used currently. Moreover, the survival of patients with metastatic breast cancer has significantly improved over the last three decades^10–12^. Furthermore, a subset of patients with metastatic breast cancer experience durable clinical remission when they are treated with intensive multidisciplinary approaches for oligometastatic lesions^13,14^. Finally, there has been a significant improvement in the diagnostic accuracy of various imaging techniques. Thus, the clinical benefit of intensive screening for distant metastasis should be reevaluated.

Retrospective analysis of the benefit of intensive screening for patients with breast cancer has major drawbacks: patients at a higher risk of developing distant metastasis may undergo imaging tests more frequently, resulting in selection bias^15,16^. Moreover, patients who undergo intensive screening may show improved post-relapse survival, as the metastatic lesions might be detected earlier, resulting in lead-time bias, and the lesions can be biologically indolent, causing length bias^17^. Accordingly, in the present study of 398 patients with breast cancer with distant metastasis, we tried to minimize selection bias by excluding all patients without distant metastasis and aimed to negate lead-time bias by defining survival as the duration between the date of initial treatment and the date of death.

## Patients and methods

### Patients

We obtained the baseline clinical data and reviewed the detailed information of patients with breast cancer who were diagnosed between January 2000 and July 2015 from our institutional database of patients with breast cancer. We included patients who developed distant metastasis after the initial recurrence-free survival (RFS) treatment. We excluded patients with synchronous or metachronous malignancies in other organs, bilateral breast cancer, male breast cancer, and recurrent breast cancer. We identified 398 patients who were initially diagnosed with non-metastatic, resectable breast cancer, received follow-up care in our institution, and eventually developed distant metastasis. From the database, we obtained the baseline characteristics and clinicopathologic information. Initial breast cancer was pathologically staged according to the 7th AJCC criteria. Hormone receptor (i.e., HR, including estrogen and/or progesterone receptors) data were collected according to immunohistochemistry findings, with positivity defined as > 1%. Human epidermal growth factor receptor type 2 (HER2) status was evaluated with anti-HER2 antibodies and/or fluorescence in situ hybridization. We also collected the data regarding the use of various imaging studies including chest radiography, bone scintigraphy, computed tomography (CT), ultrasonography (USG), magnetic resonance imaging (MRI), and fludeoxyglucose-positron emission tomography (^18^F-FDG/PET).

### Distant metastasis and screening intensity

Distant metastasis was defined as any recurrences at any sites outside the breast and regional lymph nodes. The metastatic sites included the bones, lungs, pleura, liver, brain, and distant lymph nodes; they were classified into bone, visceral (lung, pleura, liver, brain, and distant lymph node), and mixed metastases (bone and visceral) for comparison. When metastases were observed in multiple organs within 2 months of treatment, they were defined as multiple site metastases. The clinical diagnosis of distant metastasis was made after histologic confirmation of metastasis or imaging findings compatible with metastasis when biopsy was not feasible. We also reviewed the presence of symptoms associated with metastases using each patient’s medical records. Ambiguous cases such as the perception of symptoms after knowing the presence of metastasis or symptoms not associated with the site of metastases were considered asymptomatic.

**Figure 1:**
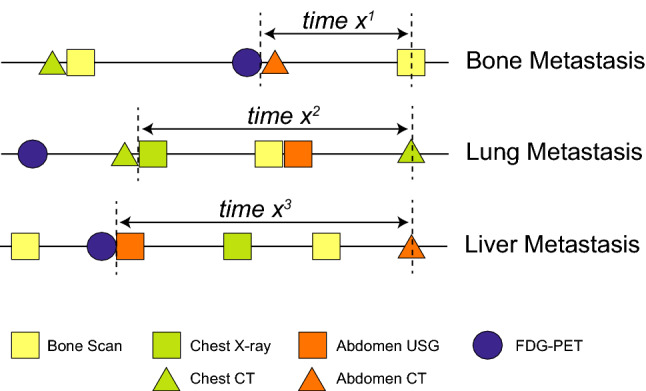
Definition of time intervals. We calculated the interval between the date of clinical diagnosis of metastasis and the date of previous imaging studies of target organs. For instance, for a patient with lung metastasis, as shown in this figure, the time interval (X^2^) was defined as the date between chest CT at diagnosis of metastasis and previous chest radiography, not including abdominal USG and abdominal CT. ^18^F-FDG-PET was allowed regardless of metastasis sites. The time intervals for patients with bone metastasis (X^1^) and those with liver metastasis (X^3^) were calculated with the same principles. USG, ultrasonography; CT, computed tomography; ^18^F-FDG-PET, fludeoxyglucose-positron emission tomography.

To assess the intensity of distant metastasis screening, we calculated the time interval between the date of clinical diagnosis of distant metastasis and the date of previous imaging examinations that targeted the organ where the metastasis developed. For example, if a patient developed bone metastasis, the screening intensity was determined considering the date of the previous bone scintigraphy or ^18^F-FDG/PET. For lung and liver metastases, the dates of chest radiography, chest CT, and 18F-FDG/PET and the dates of abdominal USG, abdominal CT, and ^18^F-FDG/PET were considered, respectively (Fig. 1). Additionally, we performed same analysis using a different definition of surveillance intensity by calculating total number of exams between the time of operation and diagnosis of distant metastasis dividing with RFS for each patient. Moreover, only tests that were conducted within 2 years before the occurrence of metastasis were analyzed separately.

### Statistical analyses

Overall survival was the time between the date of initial diagnosis and the date of death. Recurrence-free survival was the time from the date of initial diagnosis to the date of first clinical diagnosis of distant metastasis. The date of death was obtained from the Office for National Statistics of Korea. Survival analyses were performed with the Kaplan–Meier method. The log-rank test and Gehan-Breslow-Wilcoxon test were used to compare survival curves. The Cox proportional hazards regression model was used for multivariate survival analysis. Variables that showed a *P*-value < 0.05 on the log-rank or Breslow test were included in multivariate analysis. All analyses were performed using SPSS (version 22.0; SPSS, Inc.). The statistical significance was set at *P* < 0.05.

### Ethics approval and consent to participate

This study was approved by the institutional review board (IRB) of Seoul National University Hospital (SNUH; IRB No. H-1905-047-1031), and the study followed the Declaration of Helsinki and good clinical practice guidelines. All patients gave informed consent.

## Results

### Patient characteristics

We identified 398 patients with breast cancer who developed distant metastasis and who met the inclusion criteria. The mean age at the time of initial treatment was 47.6 ± 11.0 years. Almost half of the patients had stage III breast cancer initially (45.8%), and two-thirds of the patients underwent mastectomy (68.6%). The clinical characteristics of the included patients are listed in Table 1. The interval between the detection of metastatic lesions and the date of previous imaging studies for the particular organ for each individual patient is shown in Fig. 1. The median interval between the previous imaging study and the detection of metastasis was 10.5 ± 9.8 months. Our patients were classified into two groups: the intensive screening group (ISG, n = 199) and the less-intensive screening group (LSG, n = 199), with median intervals of 4.5 ± 1.6 and 16.4 ± 11.0 months, respectively. The ISG had a significantly higher incidence of neoadjuvant chemotherapy, postoperative radiotherapy, stage III disease, and previous history of locoregional recurrence, and was more likely to be diagnosed in more recent years (Table 1).

**Table 1:** Demographic and clinical characteristics of patients.

Characteristics	Total (n = 398)	ISG (n = 199)	LSG (n = 199)	P-value
Initial age (years)	47.6 ± 11.0	46.8 ± 10.5	48.3 ± 11.5	0.081
BMI (kg/m^2^)	23.5 ± 3.3	23.4 ± 3.5	23.6 ± 3.2	0.906
**Menopausal status**				0.098
Premenopausal	248 (62.3)	132 (66.3)	116 (58.3)	
Postmenopausal	150 (37.7)	67 (33.7)	87 (41.7)	
**Neoadjuvant chemotherapy**				< 0.001
Administered	98 (24.6)	64 (32.2)	34 (17.1)	
Not administered	300 (75.4)	135 (67.8)	165 (82.9)	
**Surgery type**				0.052
BCS	125 (31.4)	72 (36.2)	53 (26.6)	
Mastectomy	273 (68.6)	127 (63.8)	146 (73.4)	
**Chemotherapy**				0.177
Administered	359 (90.2)	175 (87.9)	184 (92.5)	
Not administered	39 (9.8)	24 (12.1)	15 (7.5)	
**Radiotherapy**				0.002
Administered	257 (64.6)	143 (71.9)	114 (57.3)	
Not administered	141 (35.4)	56 (28.1)	85 (42.7)	
**TNM stage**^**a**^				0.020
I	34 (9.0)	10 (5.4)	24 (12.5)	
II	171 (45.2)	81 (43.5)	90 (46.9)	
III	173 (45.8)	95 (51.1)	78 (40.6)	
**Histologic grade**				0.937
I–II	120 (32.3)	59 (32.1)	61 (32.4)	
III	252 (67.7)	125 (67.9)	127 (67.6)	
**Lymphovascular invasion**				0.104
Present	230 (57.8)	123 (61.8)	107 (53.8)	
Absent	168 (42.2)	76 (38.2)	92 (46.2)	
**Hormone receptor status**				0.513
Positive	195 (49.4)	94 (47.7)	101 (51.0)	
Negative	200 (50.6)	103 (52.3)	97 (49.0)	
**HER2 expression**				0.221
Positive	127 (32.8)	59 (29.6)	68 (35.8)	
Negative	260 (67.2)	138 (70.1)	122 (64.2)	
**Ki-67 index**				0.091
≥ 15%	136 (35.1)	77 (39.1)	59 (30.9)	
< 15%	252 (64.9)	120 (60.9)	132 (69.1)	
**Subtype**				0.351
Luminal A	121 (31.4)	62 (31.6)	59 (31.2)	
Luminal B	67 (17.4)	31 (15.8)	36 (19.0)	
Her-2 enriched	91 (23.6)	42 (21.4)	49 (25.9)	
TNBC	106 (27.5)	61 (31.1)	45 (23.8)	
**Year of metastasis**				0.001
2001–2007	226 (56.8)	97 (48.7)	129 (64.8)	
After 2008	172 (43.2)	102 (51.3)	70 (35.2)	
**Previous local recurrence**				0.008
Present	116 (29.1)	70 (35.2)	46 (23.1)	
Absent	325 (70.9)	129 (64.8)	153 (76.9)	
Time interval between examinations (months)	10.5 ± 9.8	4.5 ± 1.6	16.4 ± 11.0	< 0.001

Data are number of patients and percent (%) or mean ± standard deviation.

*ISG* intensive surveillance group, *LSG* less-intensive surveillance group, *BMI* body mass index, *BCS* breast-conserving surgery, *HER2* human epidermal growth factor receptor 2, *TNBC* triple-negative breast cancer.

^a^Stratified according to the American Joint Committee on Cancer (AJCC) 7^th^ TNM stage.

### Survival outcomes and screening intensity

**Figure 2:**
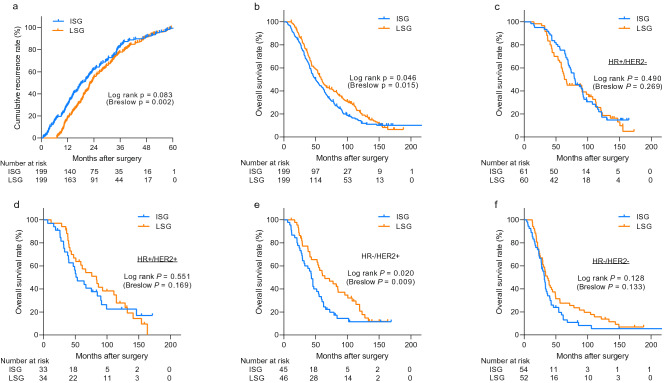
Kaplan–Meier curves showing recurrence-free survival and overall survival of all patients. The Kaplan–Meier curves show recurrence-free survival **(a)** and overall survival **(b–f)**. The survival curves for all 398 patients **(b)** and for patients stratified according to the hormone receptor and HER2-overexpression status **(c–f)** are shown. Among all, 13 patients were unable to obtain the data of subtypes. P-values were calculated by using the log-rank test along with Gehan–Breslow–Wilcoxon tests (in the parenthesis). *ISG* intensive surveillance group, *LSG* less-intensive surveillance group, *HR* hormone receptor, *HER2* human epidermal growth factor receptor type 2.

The distant-metastasis free survival of the 398 patients with breast cancer according to the frequency of imaging studies is shown in Fig. 2a. Patients in the ISG had a significantly shorter distant-metastasis free survival especially in the early phase of follow-up than in the LSG (log rank p = 0.083, Breslow p = 0.002). The LSG had a significantly higher overall survival (log rank p = 0.046, Breslow p = 0.015, Fig. 2b). However, after adjusting for other prognostic factors, multivariate Cox regression analysis revealed no significant difference in overall survival between the two groups (hazard ratio [HR] = 1.21, 95% confidence interval [CI]: 0.95–1.54; p = 0.124; Table 2). The initial N stage, hormone receptor status, Ki-67 expression level, history of previous locoregional recurrence, presence of symptoms at the diagnosis of distant metastasis, and metastatic site remained independent factors predicting overall survival. Additionally, we used different definition of surveillance intensity based on the number of all exams during the RFS. The survival analysis showed similar results including that of the Cox-regression analysis (Supplementary Fig. 1). To further minimize the effect of the confounding variables, we conducted propensity score matching incorporating initial N stage, hormone receptor status, Ki-67 expression level, history of previous locoregional recurrence, presence of symptoms and metastatic sites. Based on propensity score matching, a total of 159 pairs of patients was included in the survival analysis. There was no difference in overall survival between matched LSG and ISG groups (log rank p = 0.264, Breslow p = 0.129, Supplementary Fig. 2a).

**Table 2:** Clinicopathologic features affecting post-operative overall survival.

	Univariate analysis		Multivariate analysis	
	P-value*	HR (95% CI)	P-value	HR (95% CI)
**Initial age (years)**	0.746 (0.728)		–	–
< 40		Ref		
≥ 40		0.96 (0.75–1.23)		
**BMI (kg/m**^**2**^**)**	0.539 (0.985)		–	–
< 25.0		Ref		
≥ 25.0		1.08 (0.85–1.36)		
Post-menopausal status	0.750 (0.968)	1.04 (0.83–1.29)	–	–
Neoadjuvant chemotherapy	0.090 (0.004)		0.689	
Administered		Ref		Ref
Not administered		0.80 (0.62–1.04)		1.06 (0.78–1.45)
Surgery type	0.130 (0.277)	–	–	–
Breast-conserving		Ref		
Mastectomy		1.20 (0.95–1.52)		
**Chemotherapy**	0.645 (0.999)		–	–
Administered		Ref		
Not administered		0.91 (0.61–1.36)		
**Radiotherapy**	0.489 (0.882)		–	–
Administered		Ref		
Not administered		0.92 (0.74–1.16)		
**T stage**	0.028 (0.108)		0.372	
I		Ref		Ref
II		1.29 (0.96–1.74)		1.23 (0.89–1.71)
III–IV		1.51 (1.04–2.19)		1.33 (0.86–2.05)
**N stage**	< 0.001 (< 0.001)		0.014	
0		Ref		Ref
I		1.19 (0.89–1.60)		1.17 (0.85–1.61)
II		1.47 (1.07–2.01)		1.60 (1.13–2.27)
III		1.88 (1.38–2.56)		1.63 (1.13–2.33)
**Histologic grade**	0.067 (< 0.001)		0.988	
I–II		Ref		Ref
III		1.25 (0.99–1.58)		1.00 (0.77–1.31)
Lymphovascular invasion	0.073 (0.161)	1.22 (0.98–1.52)	-	-
Hormone receptor negativity	< 0.001 (< 0.001)	1.72 (1.38–2.13)	0.002	1.48 (1.15–1.91)
HER2 expression	0.993 (0.681)	1.00 (0.79–1.26)	-	-
High Ki-67 index	0.002 (< 0.001)	1.45 (1.15–1.82)	0.025	1.34 (1.04–1.74)
**Year of metastasis**	0.004 (0.001)		0.052	
2000–2007		Ref		Ref
After 2008		0.73 (0.58–0.91)		0.78 (0.61–1.00)
Previous local recurrence	< 0.001 (0.001)	1.69 (1.34–2.13)	0.110	1.25 (0.95–1.65)
**Site of first metastasis**	< 0.001 (0.001)		0.009	
Bones		Ref		Ref
Visceral		1.25 (0.94–1.67)		1.04 (0.75–1.44)
Mixed		2.05 (1.54–2.73)		1.53 (1.10–2.12)
Symptoms present	< 0.001 (< 0.001)	2.11 (1.70–2.63)	< 0.001	1.66 (1.29–2.14)
Intensive surveillance	0.046 (0.015)	1.24 (1.00–1.55)	0.124	1.21 (0.95–1.54)

*HR* hazard ratio, *CI* confidence interval, *BMI* body mass index, *HER2* human epidermal growth factor receptor 2, *Ref.* reference.

*The *p* values are derived from the Log-rank test and the p values from the Breslow test are shown in the parenthesis.

We then examined the association between the screening intensity for distant metastasis and survival considering different subtypes of breast cancer. There was no significant interaction between variables including subtypes and sites of metastasis and intensity of surveillance (p > 0.05, data not shown). As shown in Fig. 2c–f and supplementary Fig. 3, the screening intensity did not affect the survival outcomes considering HR + /HER2-, HR + /HER2 + , and HR-/HER2- subtypes. However, the LSG group had significantly better overall survival than the ISG group did when the tumors were HR-/HER2 + . Nevertheless, the prognostic importance of screening intensity did not remain significant in this subgroup after adjusting for other prognostic factors by using a Cox regression model (HR = 1.47, 95% CI: 0.80–2.73; p = 0.217, Supplement Table 1).

**Figure 3:**
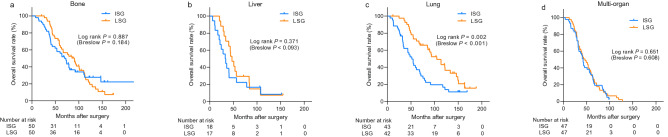
Kaplan–Meier curves showing overall survival depending on initial metastases sites. The survival curves for patients with bone **(a)**, liver **(b)** and multiple metastases **(d)** showed no significant differences between the two groups. However, the intensive surveillance group showed a significantly low overall survival among patients with lung metastasis **(c)**. *ISG* intensive surveillance group, *LSG* less-intensive surveillance group.

### Sites of metastasis, presence of symptoms, and effects of screening intensity

Among the 398 patients with distant metastasis, 220 developed distant metastasis in a single organ: 100 patients had bone metastasis, 85 had lung metastasis, and 35 had liver metastasis. The remaining 178 patients developed metastases in multiple organs. The intensity of screening did not affect the overall survival of patients who developed metastasis in the bones, liver, or multiple organs. However, the overall survival of patients whose first site of metastasis was the lungs was significantly low (Fig. 3). The screening intensity remained an independent prognostic factor of overall survival in patients with lung metastasis after adjusting for other prognostic factors (HR = 2.10, 95% CI: 1.06–4.17; p = 0.034, Supplement Table 2). However, the patients with lung metastasis in the ISG showed higher incidence of symptoms (43.9% vs. 19.5%, p = 0.018) and previous history of local recurrences (41.5% vs. 14.6%, p = 0.007).

**Figure 4:**
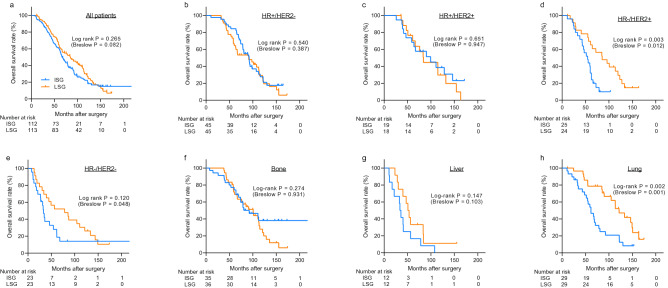
Kaplan–Meier curves showing overall survival of asymptomatic patients and subgroups. The Kaplan–Meier curves show the overall survival of 225 asymptomatic patients. Overall survival was analyzed after dividing the patients into the intensive surveillance group and less-intensive surveillance group **(a)**. Further subgroup analysis was performed according to subtypes **(b–e)** and sites of metastases **(f–h)**. Among asymptomatic patients, 3 patients were unable to obtain the data of subtypes. *ISG* intensive surveillance group, *LSG* less-intensive surveillance group, *HR* hormone receptor, *HER2* human epidermal growth factor receptor type 2.

As the presence of symptoms at the time of diagnosis might lead to the performance of imaging studies earlier than the pre-scheduled dates, patients who develop symptomatic, rapidly progressing distant metastasis are more likely to have a shorter time interval between the previous imaging studies and the diagnosis of distant metastasis. To overcome this issue, we identified 225 patients whose distant metastases were asymptomatic and who were diagnosed using screening imaging studies. As shown in Fig. 4a, we observed similar associations between the screening intensity and the survival outcomes of patients with asymptomatic distant metastasis. Also, the propensity score matching analysis for asymptomatic patients using 96 pairs of patients showed the same result (Supplementary Fig. 2b). Patients in the LSG had significantly higher overall survival when the patients had HR-/HER2 + tumors and when the first site of metastasis was the lungs (Fig. 4b–h, Supplementary Fig. 3). Among these 225 asymptomatic patients, we also observed that the screening intensity showed borderline prognostic significance in HR−/HER2− subtype (Fig. 4e). However, the interaction assessment among asymptomatic patients revealed significant interaction between both hormone receptor status (p = 0.001) and the metastasis site (p = 0.022) and the intensity of surveillance.

## Discussion

In the current study, we showed that intensive imaging during the post-treatment follow-up period was not associated with survival benefit in patients with breast cancer. We also observed that in a subset of patients, i.e., patients with lung metastasis, highly intensive screening for distant metastasis was associated with poor survival outcomes. Our data indicate that, despite the recent development in targeted therapy for patients with stage IV breast cancer, earlier detection of distant metastasis does not result in survival benefit for patients with breast cancer who developed distant metastasis.

Two randomized controlled trials have evaluated the efficacy of intensity of surveillance in patients with breast cancer. In 1994, the Interdisciplinary Group for Cancer Care Evaluation (GIVIO)^7^ randomized 1320 patients with breast cancer into intensive or clinical surveillance; they reported that treatment outcomes and quality of life were not significantly different between the groups after follow-up for 71 months. Similarly, Del Turco et al. and Palli et al.^6,18^ enrolled 1,243 patients, and showed significantly higher recurrence free survival for clinical groups, but failed to show significant difference on overall mortality at 5- and 10-years of follow-ups. The data showed the lack of survival benefit of intensive surveillance for distant metastasis; these are the basis of the current guidelines that recommend against routine imaging studies—except mammography—for asymptomatic disease-free patients with breast cancer^4,5,19^. However, these trials were conducted before the era of targeted therapies such as trastuzumab (NCT00829166) or CDK inhibitors (NCT01740427), which have significantly improved the survival of patients with stage IV breast cancer^20,21^. Moreover, the diagnostic accuracy of modern imaging studies has substantially improved since these clinical trials^22–24^. Therefore, our study included patients who were diagnosed between 2000 and 2015, and the results show that intensive surveillance still lacks any survival benefit.

Although current guidelines^4,5,19^ and systematic review^9,25^ do not recommend routine imaging for patients with breast cancer, real-word practices often involve the use of advanced imaging studies owing to the belief that earlier detection of distant metastasis may lead to improved survival ^15,16,26,27^. Moreover, diagnostic studies for distant metastasis may provide emotional support and reassurance to both the physician and patient ^6,28–30^. However, frequent visits may elevate the anxiety of breast cancer survivors ^31^, and intensive surveillance may increase false-positive results for distant metastasis that may further increase the psychological burden ^32,33^. Furthermore, Meyer et al.^34^ reported a significant association between intensive surveillance and the risk of secondary cancer or radiation-induced malignancy in the patient’s lifetime. Therefore, the decision regarding optimal surveillance after the initial treatment for breast cancer must be well-balanced after considering the advantages and disadvantages.

The current study has several limitations. This was a retrospective study from a single, high-volume institution. The retrospective nature inherently raises the possibility of selection bias. To eliminate the effect of selection bias that high-risk patients with breast cancer may undergo very intensive screening, we limited our analysis to patients who eventually developed distant metastasis. Despite our efforts of excluding patients who did not develop distant metastasis, patients with more aggressive features within the study population were more likely to undergo intensive surveillance, as indicated in our results. This selection bias may have masked the potential protective effect of intensive surveillance, because the high-risk features—such as triple-negative subtype or advanced stage at diagnosis—are associated with shorter time to death after the development of distant metastasis^12^. Therefore, our current findings require further validation by using data from a multi-institutional database or a nationwide registry. However, obtaining detailed clinical information about the type of metastasis and the use of imaging studies remain a major hurdle for such validation studies. In addition, we could not adjust for the complex information regarding the use of adjuvant systemic therapies and the response to palliative systemic treatment. Moreover, our study population included a substantial proportion of patients whose disease severity was determined by using the clinical TNM staging because they underwent neoadjuvant therapy. The potential discrepancy between the clinical stage and anatomic stage of breast cancer might have made our results more complex^35^. Finally, it is unclear why intensive surveillance was associated with worse overall survival in patients with HR−/HER2 + tumors or patients who developed lung metastasis in our study. It is possible that small number of patients in each subgroup or unknown confounding factors may have resulted this observation. Additionally, unadjusted confounding factors may have contributed to this observation. Cox-regression analysis also showed significant interaction between both hormone receptor status or metastasis sites and intensive surveillance. Unfortunately, we were not able to perform additional propensity score matching analysis due to the small number of patients in this subgroup. Alternatively, we cannot also exclude the possibility that higher anxiety caused by intensive screening may have affected the survival outcomes, because stress and anxiety promoted tumor progression in mouse models of various solid tumors including breast tumors^36–38^.

## Conclusion

The results of this retrospective study suggest the lack of any association between intensive surveillance for distant metastasis and survival benefit in asymptomatic, disease-free patients with breast cancer after their initial treatments.

## Supplementary Information

Supplementary Information.

## Data Availability

The datasets used and/or analyzed during the current study are available from the corresponding author on reasonable request.
