# Supplementary material for: Intensity of metastasis screening and survival outcomes in patients with breast cancer
Source: Sci Rep. 2021 Feb 2;11:2851. doi: 10.1038/s41598-021-82485-w (PMC7854644; doi:10.1038/s41598-021-82485-w)

**Supplemental material**

**Intensity of metastasis screening and survival outcomes in patients with breast cancer**

Jong-Ho Cheun, MD^1^, Jigwang Jung, MD^1^, Eun-Shin Lee, MD^1^, Jiyoung Rhu, MD^2^, Han-Byoel Lee, MD, PhD ^1^, Kyung-Hun Lee, MD, PhD ^3^, Tae-Yong Kim, MD, PhD ^3^, Wonshink Han, MD, PhD ^1,4^, Seock-Ah Im, MD, PhD ^3,4,5^, Dong-Young Noh, MD, PhD ^1,4^, and Hyeong-Gon Moon, MD, PhD ^1,5,*^

^1^Department of Surgery, Seoul National University College of Medicine, Seoul, South Korea ^2^Department of Surgery, College of Medicine, the Catholic University of Korea, Seoul, South Korea

^3^Department of Internal Medicine, Seoul National University College of Medicine, Seoul, South Korea

^4^Cancer Research Institute, Seoul National University

^5^Genomic Medicine Institute, Medical Research Center, Seoul National University College of Medicine, Seoul, South Korea

JH Cheun and J Jung contributed equally to this work.

^*^**Corresponding author:** Hyeong-Gon Moon, MD, PhD

Department of Surgery, Seoul National University College of Medicine, 101 Daehak-ro, Jongno-gu, Seoul, 03080 Seoul, South Korea

Tel: +82-2-2072-2634; E-mail: moonhg74@snu.ac.kr

**Table of contents**

Cox-regression analysis of postoperative overall survival among patients with HER-2 over-expression subtype Table S1

Cox-regression analysis of postoperative overall survival among patients with single lung metastasis Table S2

Kaplan-Meier curves of overall survival with various definition of intensity of surveillance Figure S1

Kaplan-Meier curves of overall survival after propensity score matching Figure S2

Subgroup analysis depending on subtypes and sites of first metastasis Figure S3

**Supplement table S1.** **Cox-regression analysis of postoperative overall survival among patients with HER-2 over-expression subtype.**

|  | *Multivariate P*-value | Hazard ratio  (±95% CI) |
| --- | --- | --- |
| Neoadjuvant chemotherapy | 0.575 | 0.80 (0.37 – 1.74) |
| T stage | 0.127 |  |
| Ⅰ |  | Ref. |
| Ⅱ |  | 1.35 (0.64 – 2.84) |
| Ⅲ-Ⅳ |  | 2.52 (0.80 – 2.73) |
| N stage | 0.172 |  |
| 0 |  | Ref. |
| Ⅰ |  | 1.57 (0.74 – 3.32) |
| Ⅱ |  | 2.17 (1.01 – 4.66) |
| Ⅲ |  | 1.07 (0.43 – 2.64) |
| Histologic grade | 0.502 |  |
| Ⅰ-Ⅱ |  | Ref. |
| Ⅲ |  | 0.79 (0.39 – 1.59) |
| High Ki-67 | 0.417 | 1.28 (0.71 – 2.30) |
| Year of metastasis | 0.557 |  |
| 2000~2007 |  | Ref. |
| 2008 ~ |  | 0.83 (0.46 – 1.53) |
| Previous local recurrence | 0.405 | 1.28 (0.71 – 2.31) |
| Site of first metastasis | 0.876 |  |
| Bone |  | Ref. |
| Visceral |  | 1.25 (0.46 – 3.41) |
| Mixed |  | 1.18 (0.41 – 3.03) |
| Symptoms present | 0.202 | 1.46 (0.82 – 2.60) |
| Intensive surveillance | 0.217 | 1.47 (0.80 – 2.73) |

**Supplement table S2. Cox-regression analysis of postoperative overall survival among patients with single lung metastasis**.

|  | *Multivariate P*-value | Hazard ratio  (±95% CI) |
| --- | --- | --- |
| Neoadjuvant chemotherapy | 0.717 | 1.19 (0.46 – 3.05) |
| T stage | 0.051 |  |
| Ⅰ |  | Ref. |
| Ⅱ |  | 2.47 (1.07 – 5.71) |
| Ⅲ-Ⅳ |  | 3.90 (1.11 – 13.72) |
| N stage | 0.019 |  |
| 0 |  | Ref. |
| Ⅰ |  | 2.19 (0.99 – 4.84) |
| Ⅱ |  | 3.89 (1.54 – 9.83) |
| Ⅲ |  | 3.01 (1.17 – 7.75) |
| Histologic grade | 0.384 |  |
| Ⅰ-Ⅱ |  | Ref. |
| Ⅲ |  | 1.42 (0.64 – 3.16) |
| Hormone receptor negative | 0.065 | 2.05 (0.96 – 4.41) |
| High Ki-67 | <0.001 | 3.47 (1.83 – 6.59) |
| Year of metastasis | 0.938 |  |
| 2000~2007 |  | Ref. |
| 2008 ~ |  | 0.97 (0.47 – 2.01) |
| Previous local recurrence | 0.128 | 2.06 (0.81 – 5.19) |
| Symptoms present | 0.233 | 1.56 (0.75 – 3.23) |
| Intensive surveillance | 0.034 | 2.10 (1.6 – 4.17) |

**Supplement figure S1. Kaplan-Meier curves of overall survival with various definition of intensity of surveillance.** When the intensity of surveillance was calculated as total number of conducted exams divided with RFS (a) and when calculated with exams conducted within just two years before diagnosis of distant metastasis (b), the results were consistent with those analyzed by the method used in this study. Additionally, Cox Proportional-Hazards model showed no significant difference between two groups by all methods (*data not shown*)


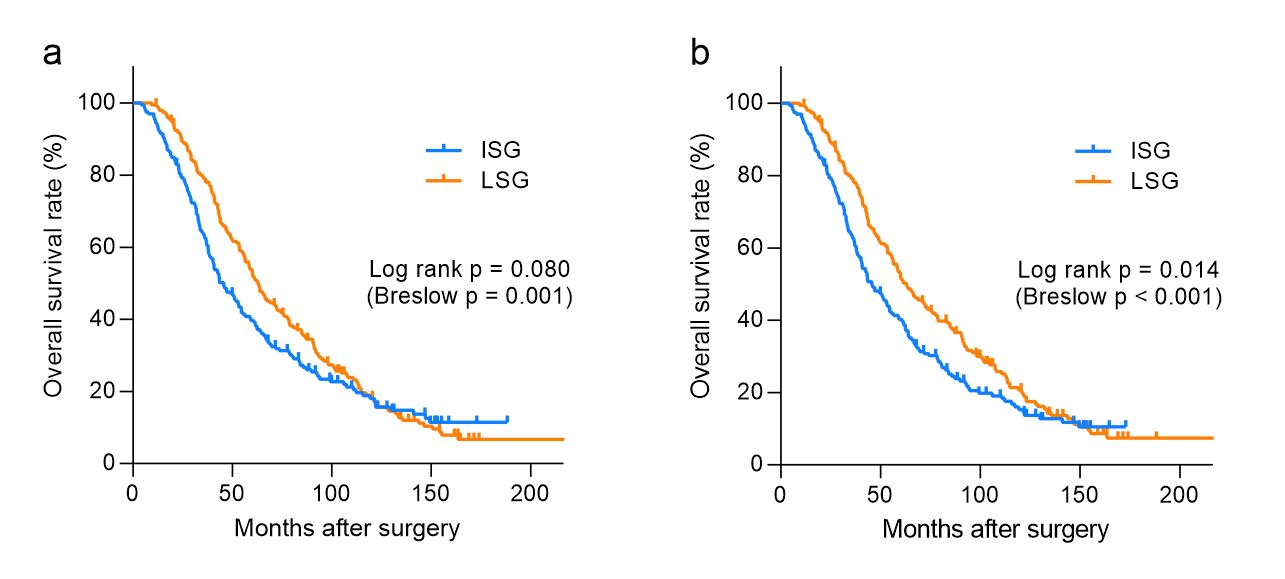


**Supplement figure S2. Kaplan-Meier curves of overall survival after propensity score matching.** To minimize confounding effects, we conducted propensity score matching with variables including initial N stage, hormone receptor status, Ki-67 expression level, history of previous locoregional recurrence, presence of symptoms at the diagnosis of distant metastasis, and metastatic site. The results showed no significant difference between two groups for all patients (159 pairs, a) and for patients with asymptomatic metastasis (96 pairs, b)


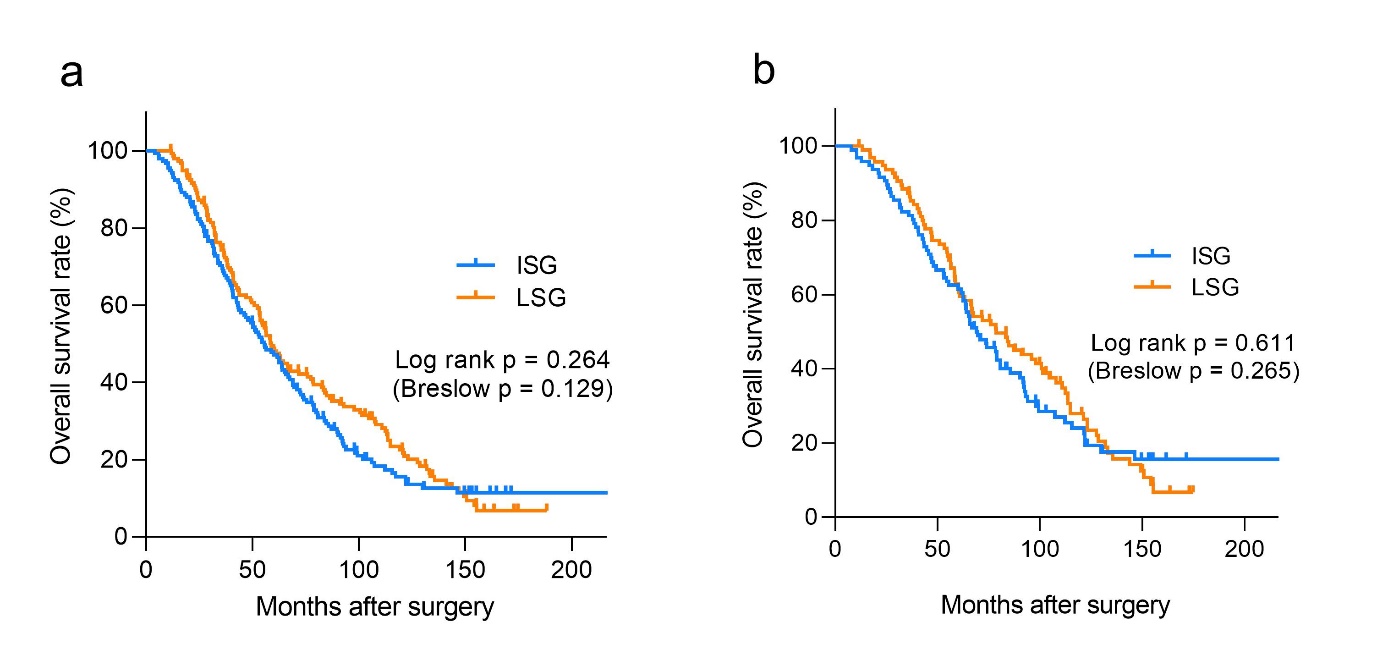


**Supplement figure S3**. **Subgroup analysis depending on subtypes and sites of first metastasis.** Patients in the LSG had significantly higher overall survival when the patients had HR-/HER2+ tumors and when the first site of metastasis was the lungs


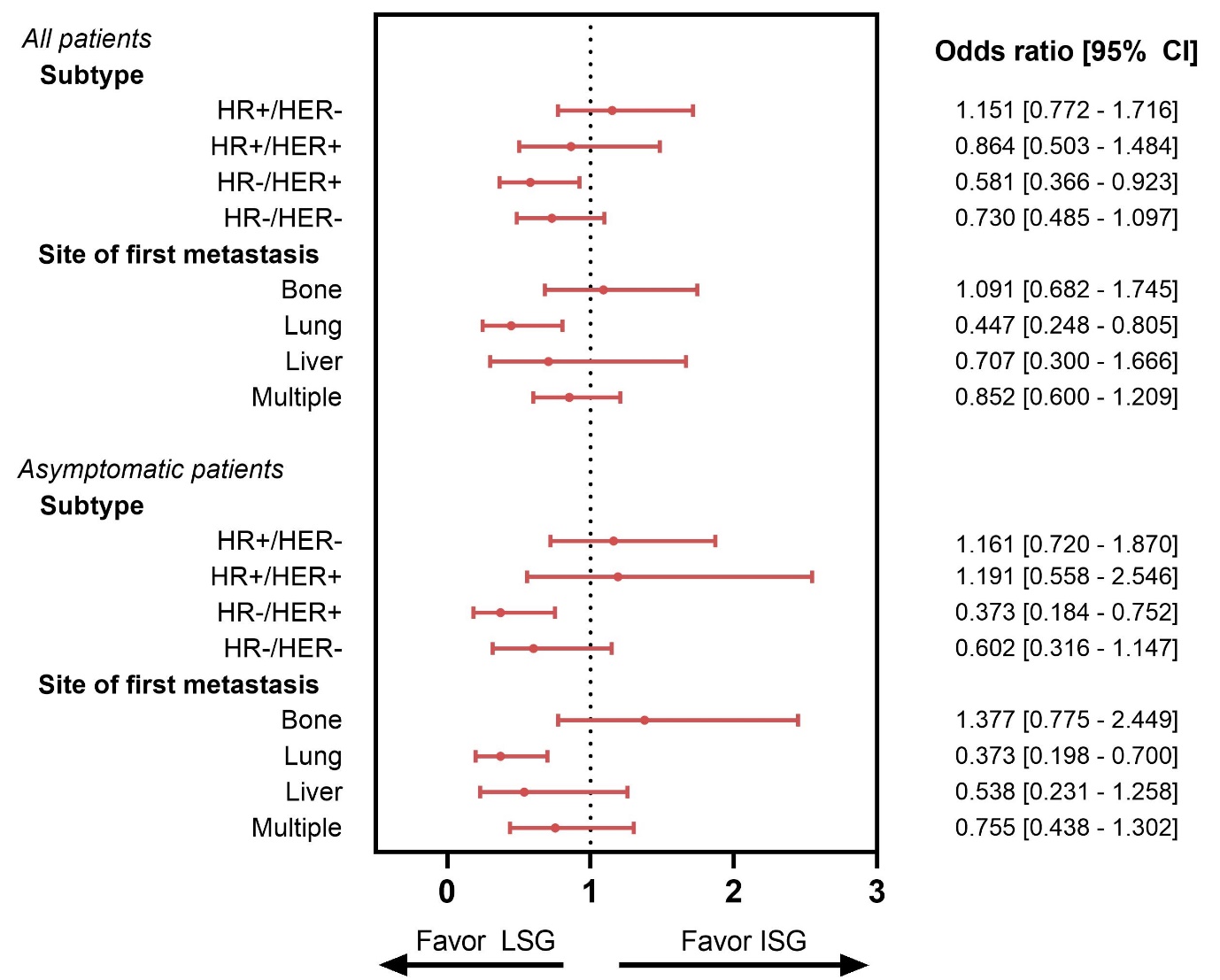

Supplement: Supplementary file 1 — Supplementary Information. [file 41598_2021_82485_MOESM1_ESM.docx]
